# Dissimilarity in radial growth and response to drought of Korshinsk peashrub (*Caragana korshinskii* Kom.) under different management practices in the western Loess Plateau

**DOI:** 10.3389/fpls.2024.1357472

**Published:** 2024-04-08

**Authors:** Cunwei Che, Mingjun Zhang, Wanmin Yang, Shengjie Wang, Yu Zhang, Lingling Liu

**Affiliations:** ^1^ College of Geography and Environmental Science, Northwest Normal University, Lanzhou, China; ^2^ Key Laboratory of Resource Environment and Sustainable Development of Oasis, Lanzhou, Gansu, China

**Keywords:** drought stress, management practice, tree-ring, *climwin* model, precipitation

## Abstract

Quantitative assessment of tree responses to the local environment can help provide scientific guidance for planted forest management. However, research on the climate-growth relationship of Korshinsk peashrub (*Caragana korshinskii* Kom.) under different land preparation and post-management (irrigation) conditions is still insufficient. In this study, we collected 223 tree-ring samples from Korshinsk peashrubs using dendroecological methods and systematically quantified the relationships between shrub growth and climatic factors under different management practices in the western Loess Plateau of China. Our findings demonstrated that drought stress caused by scarce precipitation from April to August was the primary factor limiting the growth of Korshinsk peashrubs in the northern and southern mountains of Lanzhou. The “*climwin*” climate model results showed a weak correlation between natural Korshinsk peashrub growth and drought stress, whereas planted Korshinsk peashrub under rain-fed conditions in the southern mountain was significantly (*p*<0.05) limited by drought stress from April to August. Moreover, planted Korshinsk peashrub growth under irrigated conditions in the northern mountain was limited only by drought stress in January. Drought model explained 28.9%, 38.3%, and 9.80% of the radial growth variation in Xiguoyuan (XGY), Shuibaozhan (SBZ), and Zhichagou (ZCG) sites, respectively. Artificial supplementary irrigation alleviated the limitation of drought on planted forest growth, which may be implemented for Korshinsk peashrubs planted on sunny slopes, while planted Korshinsk peashrubs under natural rain-fed conditions can be planted on shady slopes through rainwater harvesting and conservation measures such as horizontal ditches and planting holes.

## 1 Introduction

Drought is a shortage of water due to an imbalance between water supply and demand, usually caused by anomalies such as low precipitation or high temperatures. Currently, the frequency and intensity of droughts are increasing owing to climate warming (Gazol et al., 2018; Satoh et al., 2022; Wang et al., 2023). The western Loess Plateau (LP) features arid and semi-arid regions with scarce precipitation, drought, and little rain. Climate change and human activities have resulted in low forest and grass cover, sparse vegetation, and fragile ecosystems (Gong and Jin, 2004; Chen et al., 2005; Che et al., 2020). To solve these problems, the government has initiated the Grain for Green Project, which has ultimately resulted in a significant improvement in the vegetation cover of the LP (Yu et al., 2016; Xiang, 2021).

However, owing to the influence of the climatic and anthropogenic environments and the lack of research on the relationships between afforestation species and local climate (Che et al., 2022b; Li Z et al., 2023), the survival and retention rates of afforestation are very low, especially on barren mountains without irrigation. In these areas, very few stands have survived and have been preserved, growth is poor, and vegetation coverage is low, which leads to the emergence of inefficient planted forests. Currently, a soil moisture deficit due to an imbalance between precipitation and soil water remains a primary limitation to revegetation and restoration (Xiao et al., 2015; Feng et al., 2016; Xiao et al., 2019). Inefficient shrub growth after afforestation leads to the degradation and loss of ecosystem services in planted forests (Hou et al., 2019; Zhang et al., 2021, Zhang et al., 2022). Therefore, the coordination of the relationship between scarce precipitation and planted vegetation growth (so that the planted forests may reach a near-natural succession state in areas without irrigation) remains a key scientific question for the ecological restoration and stable development of vegetation in the western LP.

Tree rings can provide long-term data support for addressing these problems, and numerous researchers have used dendroecological methods to explore the climate-growth relationship of planted forests in vegetation restoration areas (Allen et al., 2010; Choat et al., 2012, Choat et al., 2018; Camarero et al., 2021; Zhang et al., 2023). In the Yangjuangou catchment of the central LP, Wei et al. (2018) compared the natural species *Armeniaca sibirica* and *Vitex negundo* var. *heterophylla* with the planted species *Robinia pseudoacacia* and *Caragana korshinskii* and found that natural tree species could adapt to the regional drought, whereas planted tree species were more sensitive to drought stress. Finally, it was suggested that introduced species be chosen carefully and natural tree species be used as the dominant species for afforestation. In the western LP, Xiao et al. (2019) analyzed the suitability of planted *Platycladus orientalis* and *Tamarix ramosissima* in arid environments and found that irrigation and rainwater harvesting measures can relieve drought stress and promote the growth of planted forests.

Korshinsk Peashrub (*Caragana korshinskii* Kom.) was the main afforested shrub species in the LP. Che et al. (Che et al., 2022b, Che et al., 2023) systematically analyzed radial growth and response to water-heat conditions based on tree-ring samples at different precipitation gradients and proposed that as precipitation increased, drought limitation on Korshinsk peashrub growth gradually decreased.

The current research on the climate-growth relationship of planted forests usually focuses on comparing natural tree species with planted tree species along different environmental gradients. This kind of research mainly relies on the classical Pearson correlation analysis, which can determine the limiting factors of tree growth but cannot calculate the explanatory power of climatic factors on tree radial growth. Furthermore, in the western LP, which is affected by dry climates, microhabitats (land preparation, irrigated, or not) have a more significant effect on the growth of planted forests than the large-scale climate systems (Liang et al., 2006; Macias-Fauria and Johnson, 2013; Chen et al., 2015). However, no systematic quantitative studies have been conducted on the climate-growth relationship of Korshinsk peashrubs under different management practices using dendroecological methods.

In view of this, here we selected Korshinsk peashrub in different land preparation and under rain-fed and irrigated conditions as our research object, and then systematically examined the responses of radial growth of planted forests to drought stress using the “*climwin*” climate response model. Considering that artificial irrigation can increase the soil water content, we hypothesized that the growth of Korshinsk peashrubs under rain-fed conditions would be more limited by drought stress than under irrigated conditions. Specifically, we answered the following two questions: (1) Are planted Korshinsk peashrubs more susceptible to drought stress than natural Korshinsk peashrubs? (2) How does artificial management affect the climate-growth relationships of Korshinsk peashrubs?

## 2 Materials and methods

### 2.1 Study area and shrub species

**Figure 1:**
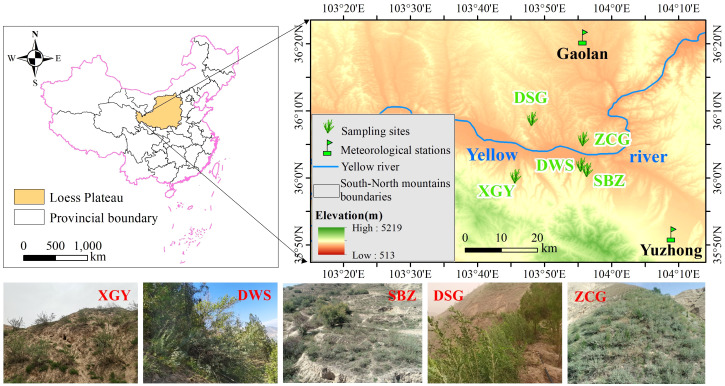
Location and landscape characteristics of Korshinsk peashrub sampling sites. (Sampling sites are abbreviated as follows: Xiguoyuan, XGY; Dawashan, DWS; Shuibaozhan, SBZ; Dashagou, DSG; Zhichagou, ZCG).

The northern and southern mountains of Lanzhou are located in the western LP, with a geographical location between 103°21′04″-104°00′38″E and 35°53′18″-36°33′56″N (**Figure 1**). Owing to the long distance from the sea, as well as the obstruction of the Qinghai-Tibet Plateau, oceanic water vapor cannot reach it directly, resulting in a temperate continental climate. The average annual temperature is 9.8°C, and the average annual precipitation is 311 mm, concentrated from July to September (accounting for over 60% of the total amount). However, annual evaporation reaches 1446 mm, which is more than five times the annual precipitation. The soil type is mainly developed on quaternary loess parent material, with sierozem as the main type (Xiao et al., 2006). Current vegetation types are mostly planted vegetation, including trees, such as *Platycladus orientalis*, *Pinus tabuliformis*, and *Robinia pseudoacacia*, as well as shrubs, such as *Caragana korshinskii*, *Hippophae rhamnoides*, and *T. ramosissima*.

Korshinsk peashrubs are deciduous shrubs. They branch extensively, with slow growth in the aboveground part during the seedling stage. The growth rate accelerates in the second year, and branching begins in the third year. They like light and do not tolerate shade. With a developed root system, they are drought-resistant and able to grow in poor soil. Therefore, they have also been introduced to the LP region (Fang et al., 2007; Wang D et al., 2020; Che et al., 2023).

In this study, the Xiguoyuan (hereinafter XGY) site represented a naturally occurring scattered forest, whereas the other sampling sites were planted forests (**Figure 1**). Shuibaozhan (hereinafter SBZ) and Dawashan (hereinafter DWS) in the southern mountain are rain-fed forests, with horizontal ditches and horizontal platforms used for land preparation, respectively. However, there is a small amount of artificial irrigation in the Dashagou (hereinafter DSG) and Zhichagou (hereinafter ZCG) in the northern mountain, with planting holes and horizontal ditches as land preparation (**Figure 1**). Additionally, the northern mountain is located on a sunny slope with more abundant sunlight, whereas the southern mountain is on a shady slope. Therefore, soil evaporation is more intense in the northern mountain than in the southern mountain (Che et al., 2020). Considering this, we set up sampling sites in both the northern and southern mountains of Lanzhou to compare how post-management affects the climate-growth relationship of Korshinsk peashrub planted forests in the northern mountain, where sunlight and evaporation are more intense.

### 2.2 Sample collection and experimental analysis

Tree-ring samples were collected following international sampling standards between August and September, 2023. Healthy and robust branches of Korshinsk peashrubs were selected from a shrubbery. A tree disc with a thickness of 3-5 cm was obtained from the base of the stem. One tree disc was collected from each shrub, and 20-30 samples were collected from each sampling site. Concurrently, growth parameters such as shrub height were investigated to compare the growth differences among shrubs in different habitats. The average values of each growth parameter were tested using a one-way ANOVA analysis (Xiao et al., 2015).

The collected tree ring samples, following the methods of dendrochronology, were smoothed and polished using sandpapers of different grit sizes. Subsequently, tree-ring widths were measured along two directions for each tree disk using a LINTAB measuring system with an accuracy of 0.001 mm. The cross-dating results and measurement quality were examined using the COFECHA program (Holmes, 1983).

In this study, the planted forests at each sampling site had the same physiological age, and each sample was measured at the pith; thus, we used the regional curve standardization method for detrending. Firstly, we fitted the regional growth curve and then divided the observed tree ring width values by the fitted values to obtain a standardized tree ring width index series for each sampling site. Finally, we employed a double-weighted mean method to obtain a standardized tree ring width index series for each sampling site (Che et al., 2023). This process was implemented using the ARSTAN program (Cook, 1985).

### 2.3 Meteorological information and data analysis

**Figure 2:**
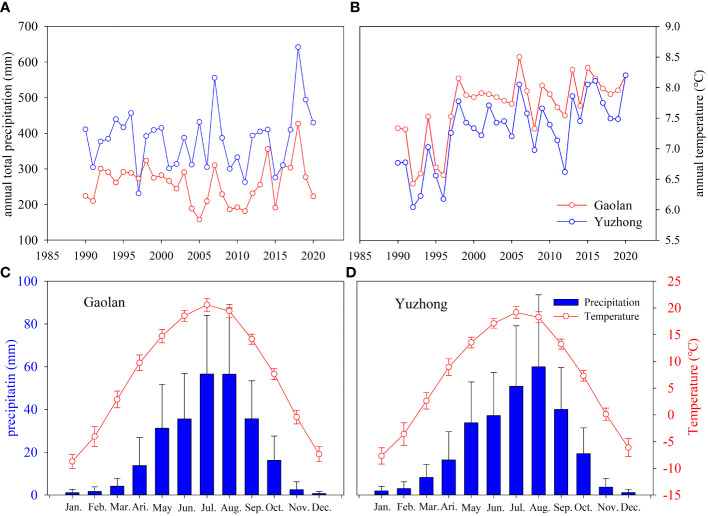
Changes in temperature and precipitation on annual and monthly scales in Gaolan and Yuzhong meteorological stations (**A**: Annual total precipitation; **B**: Annual average temperature; **C**: Monthly average Temperature and total precipitation in Gaolan; **D**: Monthly average Temperature and total precipitation in Yuzhong).

The meteorological data were obtained from the China Meteorological Data Network (http://data.cma.cn). The monthly average temperature and total monthly precipitation data in this study were obtained from the meteorological stations of Gaolan (DSG and ZCG) and Yuzhong (XGY, DWS, and SBZ), which were the nearest the sampling sites (**Figures 1**, **2**). The geographical locations of the two meteorological stations are listed in **Table 1**. Additionally, we analyzed the correlations between Korshinsk peashrub stem radial growth and drought stress using a standardized precipitation evapotranspiration index at a monthly scale (SPEI_01). The aforementioned process was implemented using the “spei” package in R (Wang B et al., 2020). Correlation analyses among tree-ring chronologies, temperature, precipitation, and SPEI_01 were performed using DENDROCLIM2002 software (Biondi and Waikul, 2004).

**Table 1:** Geographical location information of two meteorological stations.

Meteorological stations	Longitude (°E)	Latitude (°N)	Altitude (m)
Gaolan	103.93	36.35	1668.5
Yuzhong	104.15	35.86	1874.4

### 2.4 *climwin* climate response model

In this study, the “*climwin*” package in R 4.3.2 was used to identify the climate factors and windows that are most correlated with Korshinsk peashrub stem radial growth (Van et al., 2016; Gazol et al., 2018). The “*climwin*” selects the most ideal model according to the Akaike information criterion (AICc), and applies an absolute time window to analyze correlations between shrub stem radial growth and climate factors (temperature, precipitation, and SPEI_01). The Formula (1) is as follows:


(1) $${\text{AICc}}_{\text{model}}=-2\log(L)+2P+\left(\frac{2P(P+1)}{N-P-1}\right)$$

where *L* denotes the likelihood of the data in a given model, *P* denotes the number of model parameters being evaluated, and the (2P(P+1))/(N-P-1) corresponds to the small-sample correction factor.

To facilitate the comparison between models, “*climwin*” compares the AICc values of each model *i* with respect to the AICc value of a baseline model unaffected by climate. The Formula (2) is as follows:


(2) $$\Delta {\text{AICc}}_{\text{model}}i={\text{AICc}}_{\text{model}}i-{\text{AICc}}_{\text{baseline model}}$$

In our study, the lowest AIC_C_ value and highest R^2^ were selected as best model (Gazol et al., 2018; Camarero and Alvaro, 2020; Che et al., 2022a).

## 3 Results

### 3.1 Spatiotemporal patterns in stem radial growth of planted forests

**Figure 3:**
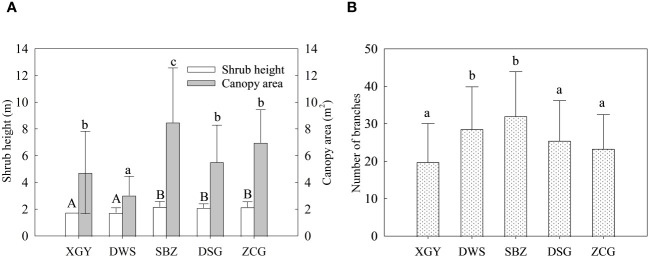
Shrub height and Canopy area **(A)**, Number of branches **(B)** of Korshinsk peashrub plant forest.

Through comparing the growth parameters of shrubs, it was found that the shrub height and canopy area of the DSG and ZCG on the northern mountain (2.11 ± 0.29 m and 2.16 ± 0.39 m; 5.52 ± 2.76 m^2^ and 6.97 ± 2.46 m^2^) were significantly (*p<* 0.05) higher than DWS site on the southern mountain (tree height and canopy area were 1.75 ± 0.35 m and 3.04 ± 1.42 m^2^) (**Figure 3A**). Moreover, by comparing the number of branches of Korshinsk peashrub on different slope aspects, it can be known that the number of branches of shrub on the sunny slope of the northern mountain (DSG and ZCG) is significantly less than the shady slope of the southern mountain (DWS and SBZ) (**Figure 3B**).

**Figure 4:**
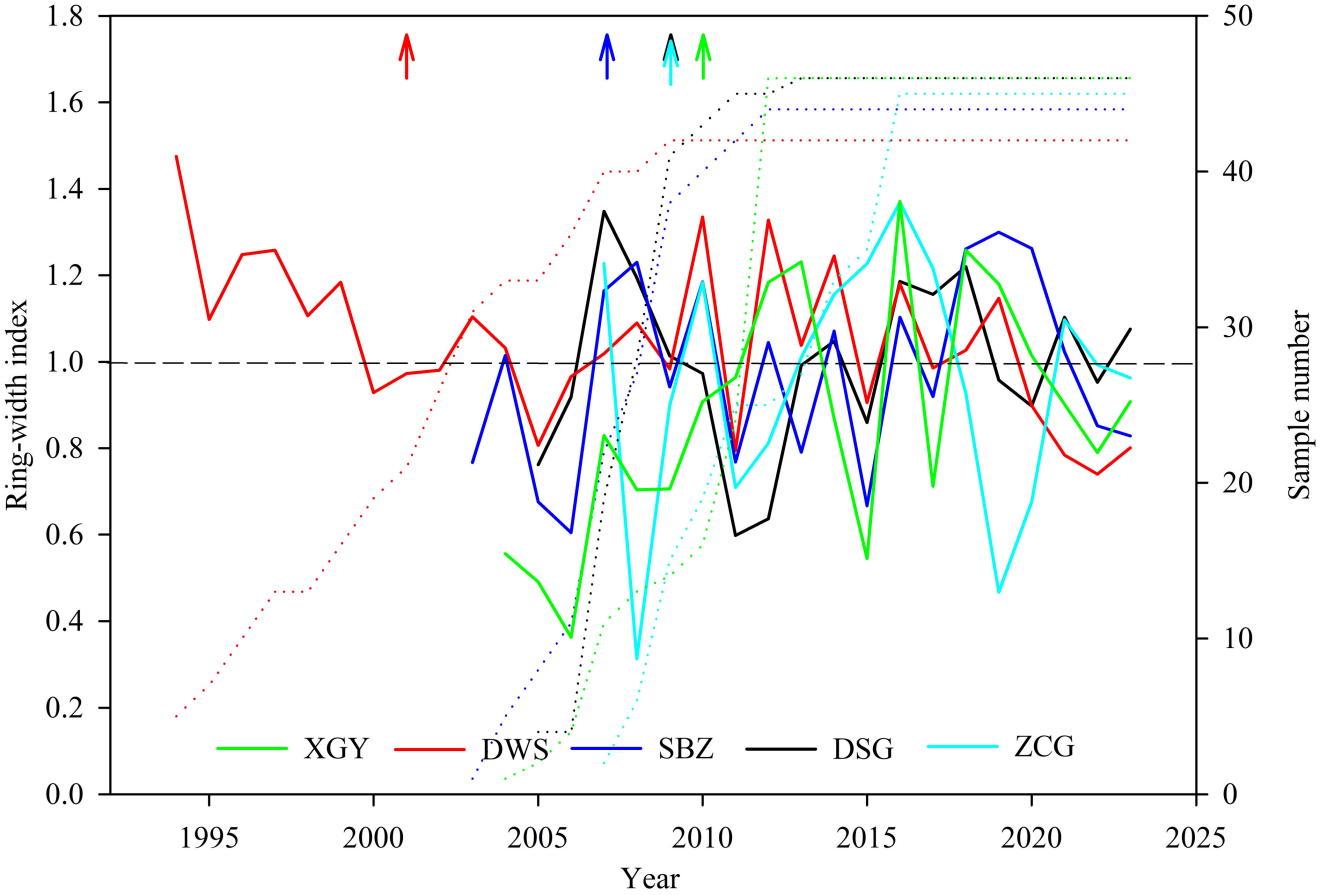
Tree-ring chronologies of Korshinsk peashrub under different management practices. (The right of the arrow indicates EPS > 0.85).

The tree-ring chronology results showed significant spatiotemporal heterogeneity under different management practices (**Figure 4**). Natural forest XGY had a chronological length of 2004-2023 and an EPS greater than 0.937. The planted forests DWS and SBZ rain-fed conditions in the southern mountain had chronological lengths of 1994-2023 and 2003-2023, with EPS values of 0.912 and 0.935 and mean sensitivities of 0.176 and 0.274, respectively, indicating that the tree-ring chronology established in this study contains abundant environmental information. The planted forests DSG and ZCG with irrigation in the northern mountain had chronological lengths of 2005-2023 and 2007-2023, and both had EPS values greater than 0.891, which met the requirements of dendroecological research (**Table 2**).

**Table 2:** Information of tree-ring chronologies and growth parameters of planted forest.

Sampling sites	XGY	DWS	SBZ	DSG	ZCG
Latitude (°N)	36.001	36.030	36.017	36.144	36.094
Longitude (°E)	103.761	103.925	103.940	103.803	103.930
Elevation (m)	1710	1769	1736	1715	1630
Chronology length	2004-2023	1994-2023	2003-2023	2005-2023	2007-2023
Sample number	46	42	44	46	45
Series intercorrelation	0.528	0.459	0.538	0.505	0.478
EPS	0.937	0.912	0.935	0.891	0.954
SNR	14.930	10.339	14.432	8.213	20.560
MS	0.299	0.176	0.274	0.194	0.374
Tree height (m)	1.77 ± 0.47	1.75 ± 0.35	2.19 ± 0.38	2.11 ± 0.29	2.16 ± 0.39
Canopy area	4.74 ± 3.08	3.04 ± 1.42	8.47 ± 4.09	5.52 ± 2.76	6.97 ± 2.46
Land preparation	natural distribution	horizontal platform	horizontal ditch	planting hole	horizontal ditch
Planting density (hm^−2^)	/	2000	1750	/	2600

MS, mean sensitivity; EPS, expressed population signal; SNR, signal-to-noise ratio.

### 3.2 The dissimilarity in response of stem radial growth to drought under different management practices

**Figure 5:**
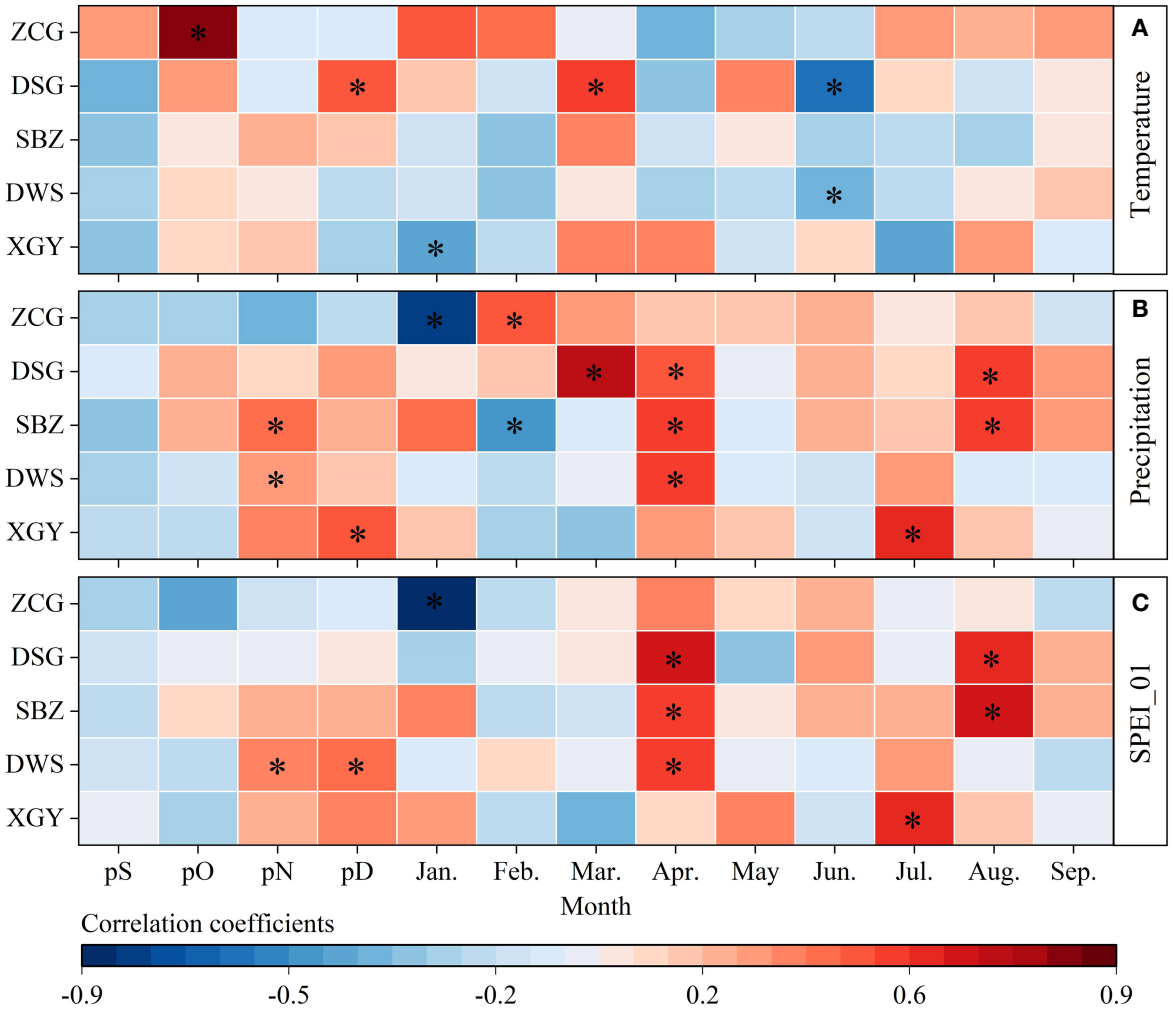
Correlation coefficients of tree-ring chronologies with temperature **(A)**, precipitation **(B)**, and SPEI_01 **(C)** from previous September (pS) to current September (Sep.).

Correlations between the stem radial growth and temperature were weak at all sampling sites (**Figure 5A**). Specifically, growth was significantly (*p*< 0.05) positively correlated with temperature in March (DSG), and negatively correlated with temperature in June (DWS and DSG) (**Figure 5A**). In addition, the Korshinsk peashrub tree-ring chronology of the natural forest XGY showed a significant positive correlation with precipitation in December and July. The planted forests DWS and SBZ under rain-fed conditions showed a significant positive correlation with precipitation in November and April (DWS and SBZ), and August (SBZ). Moreover, under irrigated conditions in the northern mountain, DSG tree-ring chronology was significantly positively correlated with precipitation in current March-April and August, whereas in ZCG, there was a significant positive correlation between tree-ring chronology and precipitation in February (**Figure 5B**).

The stem radial growth of Korshinsk peashrubs was significantly negatively correlated with temperature and positively correlated with precipitation, indicating that Korshinsk peashrub growth was significantly limited by drought stress. Therefore, we analyzed the correlations between tree-ring chronologies and SPEI_01 under different management practices (**Figure 5C**). The results showed that for XGY Korshinsk peashrubs under natural conditions, there was only a significant positive correlation between tree-ring chronology and SPEI_01 in July. Under rain-fed conditions in the DWS and SBZ, Korshinsk peashrub growth was significantly and positively correlated with SPEI_01 in the previous November-December (DWS), April (DWS and SBZ), and August (SBZ). Under irrigation conditions in DSG and ZCG, a weak correlation was observed between chronology and SPEI_01. Specifically, the DSG tree-ring chronology showed a significant positive correlation with SPEI_01 in April and August, whereas the ZCG showed a negative correlation in January (**Figure 5C**).

### 3.3 The differential contribution power of climatic factors to radial growth of shrub

The results of the “*climwin*” model demonstrated that the climate window for both precipitation and drought models of natural Korshinsk peashrub (XGY) was July. The explanatory amounts for radial growth reached 27.7% and 28.9%, respectively, whereas the effect of temperature on growth was relatively small, with a climate window from March to June and an explanatory power of 14.2%, which did not pass the significance test (*p*< 0.05; **Table 3**).

**Table 3:** Relationship between tree-ring chronology and climatic factors based on the “*climwin*” climate response model.

Sampling sites	Climate variable	Linear model		Linear model using K-fold cross-variation			
		Climate window	ΔAICc	R^2^	Climate window	ΔAICc	R^2^
XGY	Temperature	March–June	0.38	0.142	May–August	-2.57	0.021
	Precipitation	July–July	-2.53	**0.277**	August–October	-2.92	**0.277**
	SPEI_01	July–July	-2.82	**0.289**	February–July	-4.04	**0.289**
DWS	Temperature	June–June	-4.31	**0.224**	May–July	-3.07	**0.159**
	Precipitation	August–December	-1.40	0.135	July–July	-2.42	0.053
	SPEI_01	April–April	-4.54	**0.230**	February–October	-2.00	0.060
SBZ	Temperature	September–November	-0.74	0.183	April–September	-2.62	0.052
	Precipitation	August–October	-5.27	**0.365**	August–October	-2.78	0.174
	SPEI_01	August–October	-7.01	**0.423**	April–September	-3.56	**0.383**
DSG	Temperature	June–June	-3.29	**0.328**	May–December	-3.87	**0.277**
	Precipitation	August–October	-8.17	**0.504**	June–August	-3.30	**0.361**
	SPEI_01	August–October	-9.16	**0.534**	August–October	-3.20	**0.534**
ZCG	Temperature	January–December	-1.42	0.286	September–December	-3.00	0.137
	Precipitation	January–January	-6.81	**0.514**	April–April	-3.24	0.060
	SPEI_01	January–January	-8.59	**0.572**	January–January	-3.23	0.098

Significant (p< 0.05) R^2^ values are shown in bold characters.

In the rain-fed conditions of the DWS and SBZ sampling sites in the southern mountain of Lanzhou, the correlation between shrub growth and temperature was relatively weak, but was significantly (*p<* 0.05) limited by drought stress. Climate windows occurred in April and August-October, explaining 23.0% and 42.3% of growth variability, respectively (**Table 3**). Additionally, precipitation had a significant limiting effect on SBZ Korshinsk peashrub growth, although its explanatory power (36.5%) was weaker than that of drought stress. Under the irrigation conditions of the DSG and ZCG sampling sites in the northern mountain, both the precipitation and drought models passed the significance test at *p<* 0.05. Nonetheless, climate windows occurred at the end and before the beginning of the growing season (August-October and January), that is, the non-growing season (**Table 3**).

**Figure 6:**
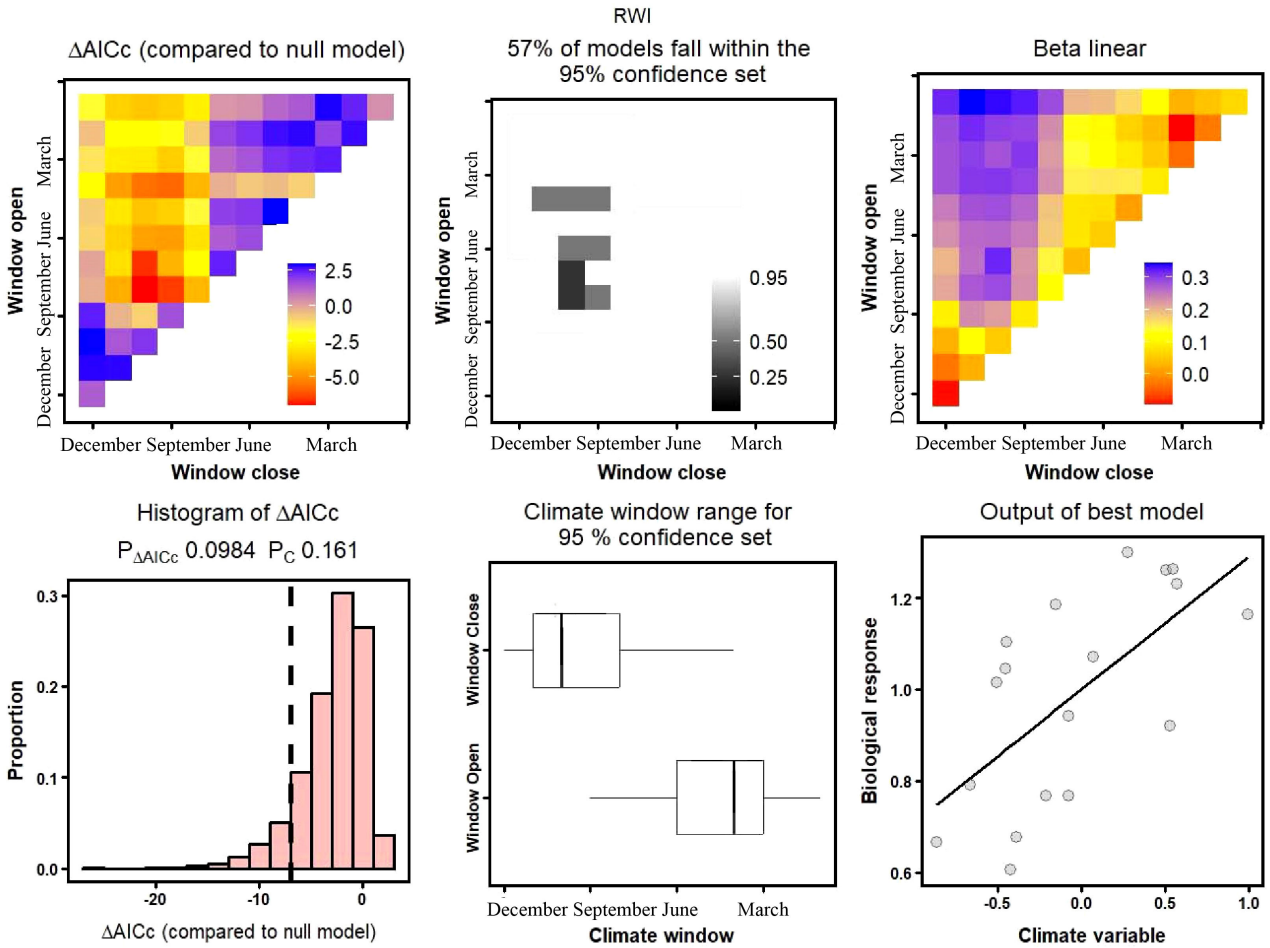
Relationship between SBZ tree-ring chronology and precipitation based on “*climwin*” climate response model. (SBZ, Shuibaozhan).

The results obtained from K-fold cross-validation and randomization tests showed that the XGY natural Korshinsk peashrub, both the precipitation and drought model were statistically significance at the 0.05 level. Nevertheless, compared to the unvalidated model, the climate windows were longer, specifically from August to October and February to July, with explanatory powers of 27.7% and 28.9%, respectively (**Table 3**). For the DWS Korshinsk peashrub in the southern mountain, the temperature model passed the significance test at *p*< 0.05 after cross-validation, and the maximum amount of growth variability (R^2^) explained by temperature was 15.9%. However, the models for precipitation and drought stress did not pass the significance test at *p<* 0.05. For the SBZ Korshinsk peashrub, the growth limitations imposed by temperature and precipitation were relatively weak. Nonetheless, the drought model passed the significance test at *p*< 0.05, with a climate window from April to September and an explanatory variable of 38.3% for radial growth (**Figure 6**).

**Figure 7:**
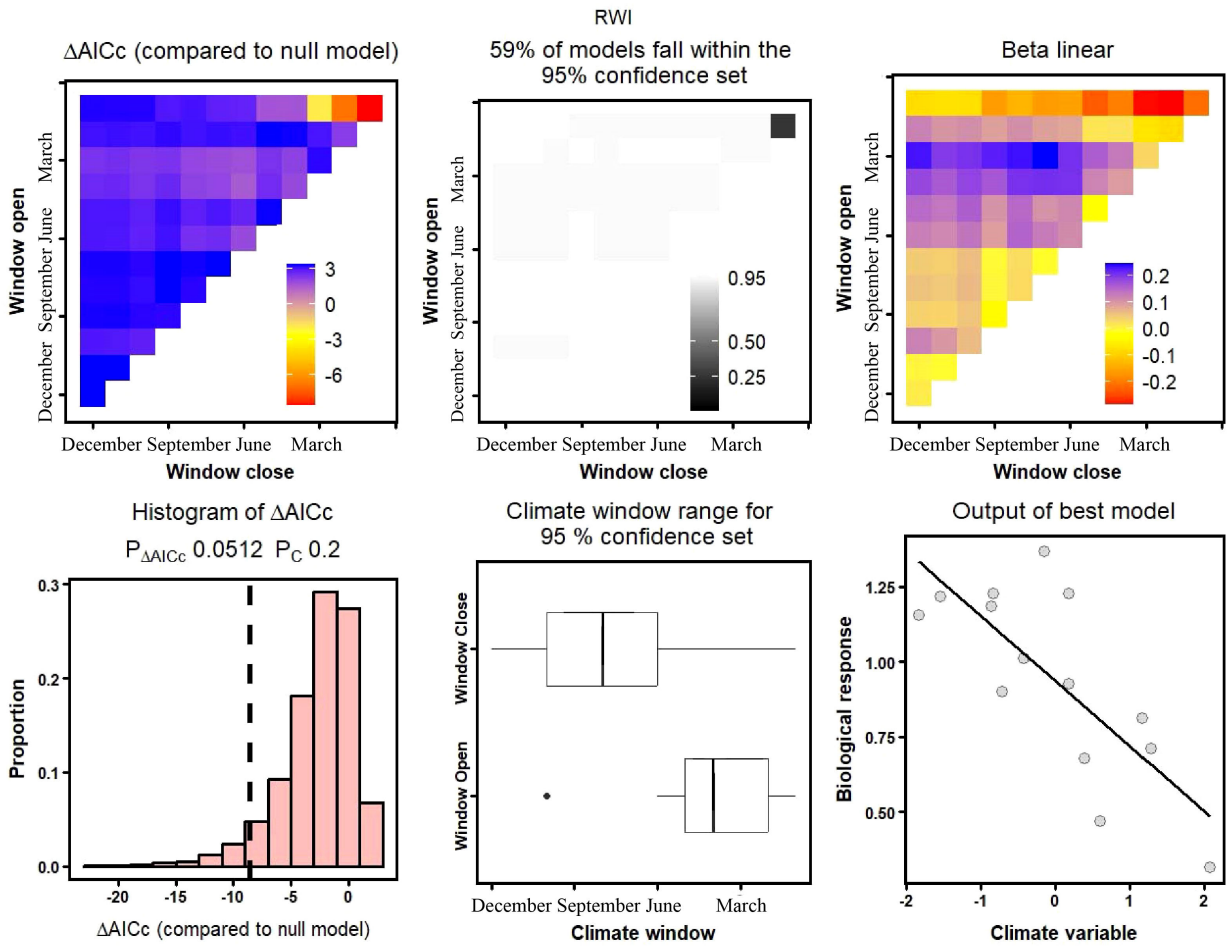
Relationship between ZCG tree-ring chronology and precipitation based on “*climwin*” climate response model. (ZCG, Zhichagou).

In comparison, for the ZCG site under irrigated conditions in the northern mountain, no significant correlation was found between temperature, precipitation, drought stress, and radial growth (**Figure 7**). However, for the DSG Korshinsk peashrub, the temperature, precipitation, and drought models all passed the significance test at *p<* 0.05, with climate windows from May to December, June to August, and August to October, explaining 27.7%, 36.1%, and 53.4% of the radial growth variability, respectively (**Table 3**). These results also confirmed the conclusions of the Pearson correlation analysis, which indicated that drought stress caused by scarce precipitation during the growing season was the primary limiting factor for Korshinsk peashrub growth, whereas the limitation imposed by temperature was relatively weak.

## 4 Discussion

### 4.1 The physiological mechanisms of radial growth climate response in planted forests

In our study, planted Korshinsk peashrub growth under different management practices showed a significant negative correlation with the temperature in June (DSG and DWS) and a significant positive correlation with precipitation and SPEI_01 in July and August (DSG, SBZ, and XGY). High temperatures increase leaf transpiration, which in turn leads to the closure of leaf stomata, weakened photosynthesis, and a reduced capacity of plants to synthesis organic matter. However, respiration continues to consume nutrients required by shrubs (Wenzel et al., 2016; Wei et al., 2018). Drought-induced water stress remains a primary limiting factor for tree growth in arid regions (Wang B et al., 2020; Wang and Yang, 2021; Wang et al., 2021; Che et al., 2023). Moreover, the growth of planted Korshinsk peashrub under irrigated conditions in the northern mountain was significantly and positively correlated with drought stress in November-December. As the tree rings have stopped growing at this time, drought may have no physiological value for shrub radial growth (Xiao et al., 2015).

In addition, the study found that under different management practices, natural Korshinsk peashrub growth in XGY showed a positive correlation with drought only in July, indicating that natural Korshinsk peashrubs adapted to the local environment. Nevertheless, when comparing the northern mountain with stronger sunlight and evaporation than the southern mountain, we found that Korshinsk peashrub growth on the northern mountain was less limited by drought stress compared to that on the southern mountain, mainly because of artificial irrigation in the northern mountain. Although the amount of irrigation is much less than the amount of precipitation, owing to geographical and other factors (Xiao et al., 2019), it can still alleviate the limitation of drought on shrub growth. As a result, Korshinsk peashrub growth in the northern mountain was limited by drought stress during the non-growing season. Moreover, owing to artificial irrigation during the growing season, the shrub height and canopy area of the northern mountain were significantly higher than those of the southern mountain. Furthermore, the physiological age of shrubs in the northern mountain was lower than in the southern mountain. These results also support the hypothesis mentioned in the introduction of this study that artificial irrigation can alleviate the limitations of drought on Korshinsk peashrub growth.

Finally, the number of branches of Korshinsk peashrub on the sunny slope of the northern mountain was significantly lower than that on the southern mountain. Considering that Korshinsk peashrubs generally start branching in the third year and uses water conservation measures such as horizontal ditches and planting holes, it can be concluded that a shady slope is more conducive to Korshinsk peashrub growth than a sunny slope.

### 4.2 Shady slopes and artificial irrigation are favorable site conditions and nurturing measures for planted forest growth

The study found that both the Pearson correlation analysis and “*climwin*” climate response model indicated that in natural rain-fed conditions of Korshinsk peashrub in SBZ, the growth was limited by drought stress from April to October, while growth in artificial irrigation conditions in DSG was limited by drought stress from August to October (the drought model explained 38.3% and 53.4% of the growth variance, respectively). These findings confirm that post-management measures involving artificial irrigation can alleviate the limitations of drought stress on Korshinsk peashrub growth (Miller et al., 2001; Xiao et al., 2019). Additionally, when comparing the mean sensitivity of tree-ring chronologies at different sampling sites, the mean sensitivity of Korshinsk peashrub in the SBZ under rain-fed conditions was 0.274, and that of artificially irrigated DSG was 0.194 (**Table 2**). Moreover, the Korshinsk peashrubs in the SBZ experienced a longer climate window of drought stress than those in the DSG, indicating that the growth of planted Korshinsk peashrubs under naturally rain-fed conditions is more susceptible to climatic factors than under artificial irrigation conditions.

However, considering that artificial irrigation requires significant labor, material, and financial resources, especially in arid climate regions with scarce precipitation in the western LP, apart from areas with concentrated human activities, such as parks and green spaces where irrigation facilities can be constructed, most areas still rely on natural precipitation (Song et al., 2006; Zhang et al., 2014; Xiao et al., 2019). Therefore, the growth of Korshinsk peashrub planted forests will still rely on the maintenance and effective utilization of natural precipitation in the future (Xiao et al., 2019; Zhang et al., 2021; Che et al., 2023).

### 4.3 The afforestation methods provide insights into the management of planted forests

In this study, we found that under similar conditions, such as physiological age and elevation, although the shrub height and canopy area of natural forest XGY were lower than those of planted forests, there was a poor correlation between tree-ring chronology and SPEI_01, suggesting that natural Korshinsk peashrubs have adapted to arid environments through long-term evolutionary processes (Wei et al., 2018). Additionally, the growth of planted Korshinsk peashrubs is primarily limited by precipitation during the growing season. With the exacerbation of global warming, the expansion of arid areas, and the accelerated frequency and magnitude of extreme drought events, this trend is expected to persist in the future (Dai, 2013; Trenberth et al., 2014; Gazol et al., 2018; Wang et al., 2023). Therefore, future management of planted forests in the northern and southern of Lanzhou should focus on the effective utilization of precipitation resources, the forestry sector must implement appropriate measures to collect and utilize limited rainwater.

In the northern and southern mountains of Lanzhou, where evaporation is much greater than precipitation, afforestation can be carried out through the “three waters” method, including horizontal ditches or planting holes to land preparation, thus increasing the infiltration capacity of water into the soil, as demonstrated in this study’s SBZ and ZCG sites. Additionally, in areas with suitable conditions, the use of plastic mulch can help retain limited precipitation resources, and seeding during the rainy season can promote seed germination and growth (Song et al., 2006; Zhang et al., 2014; Li et al., 2018). These measures will help maintain a long-term dynamic balance between the water supply and demand of planted forests in the western LP, bringing them to near-natural succession, thereby maintaining the ecosystem services of planted forests.

However, it should be noted that the western LP exhibits significant spatial heterogeneity in terms of topography, vegetation, and other factors. Specific management methods should be determined based on the local climate, microenvironment, and the climate-growth relationships of planted forests, and should be adjusted and optimized according to specific situations in a timely manner.

## 5 Conclusions

This study found that scarce precipitation from April to August was the primary limiting factor for Korshinsk peashrub growth, whereas temperature had a smaller impact on growth. Specifically, the growth of natural Korshinsk peashrubs was less influenced by drought, whereas planted Korshinsk peashrubs under rainfed conditions showed a significant positive correlation with drought stress during the growing season. In contrast, under irrigated conditions, these positive correlations were observed during the non-growing season. Moreover, on different slope aspects, the shady slope was more suitable for Korshinsk peashrub growth compared to the sunny slope. Late-stage supplementary irrigation measures can alleviate growth limitations caused by drought stress. However, considering the natural conditions in arid regions, artificial irrigation may require substantial human and financial resources. Therefore, effective utilization of existing precipitation resources is necessary for vegetation restoration in the western LP. Measures, such as horizontal ditches and planting holes, should be implemented to harvest rain and conserve moisture, and afforestation should be conducted on shady slopes to maximize the ecosystem services provided by planted forests.

## Data availability statement

The raw data supporting the conclusions of this article will be made available by the authors, without undue reservation.

## Author contributions

CC: Conceptualization, Methodology, Writing – original draft, Writing – review & editing. MZ: Funding acquisition, Investigation, Resources, Validation, Writing – review & editing. WY: Data curation, Software, Writing – review & editing. SW: Resources, Software, Writing – review & editing. YZ: Data curation, Writing – review & editing. LL: Investigation, Resources, Writing – review & editing.
